# Wearable Smart Contact Lenses for Continual Glucose Monitoring: A Review

**DOI:** 10.3389/fmed.2022.858784

**Published:** 2022-04-04

**Authors:** Mohamed Elsherif, Rosalia Moreddu, Fahad Alam, Ahmed E. Salih, Israr Ahmed, Haider Butt

**Affiliations:** ^1^Department of Mechanical Engineering, Khalifa University of Science and Engineering, Abu Dhabi, United Arab Emirates; ^2^Istituto Italiano di Tecnologia, Genoa, Italy

**Keywords:** contact lenses, glucose sensors, enzymes, phenylboronic acid, fluorescence, light diffraction

## Abstract

Diabetes mellitus is a chronic disease requiring a careful management to prevent its collateral complications, such as cardiovascular and Alzheimer's diseases, retinopathy, nephropathy, foot and hearing impairment, and neuropathy. Self-monitoring of blood glucose at point-of-care settings is an established practice for diabetic patients. However, current technologies for glucose monitoring are invasive, costly, and only provide single snapshots for a widely varying parameter. On the other hand, tears are a source of physiological information that mirror the health state of an individual by expressing different concentrations of metabolites, enzymes, vitamins, salts, and proteins. Therefore, the eyes may be exploited as a sensing site with substantial diagnostic potential. Contact lens sensors represent a viable route for targeting minimally-invasive monitoring of disease onset and progression. Particularly, glucose concentration in tears may be used as a surrogate to estimate blood glucose levels. Extensive research efforts recently have been devoted to develop smart contact lenses for continual glucose detection. The latest advances in the field are reviewed herein. Sensing technologies are described, compared, and the associated challenges are critically discussed.

## Introduction

Recent years have seen an increasing interest in improving the quality of human life, with considerable attention given to the development of reliable physiological monitoring systems. Wearable biosensors represent an important slice of the subject, due to their non-invasiveness and the potential to achieve continuous monitoring of electrical parameters and body fluid composition. Physical vital signs include body temperature, heart rate, heart pulse; body fluids analysis gives information on metabolites concentrations such as glucose, lactate, and alcohol. The most common collection sites are epidermis, mouth, and eyes (1–3). Among the various body parts, the eyes have a significant potential as sensing sites for monitoring biological signals. Glucose monitoring is relevant for a broad range of analyses, from nutritional monitoring to diabetes care.

Diabetes mellitus is a metabolic disease characterized by rising blood glucose levels. Long-term consequences of high glucose levels may cause complications such as microvascular disease, increasing the risk of stroke or ischemia, heart disease, peripheral vascular disease, retinopathy, nephropathy, and neuropathy. According to the World Health Organization (WHO), more than 220 million people worldwide suffer from diabetes. In 2012, diabetes was the tacit cause of death for approximately 1.5 million individuals, and this figure is estimated to be doubled by 2030 (4). Early diagnosis and tight control of blood glucose levels are crucial to avoid the short and long-term complications of diabetes (5). Continuous monitoring of glucose is essential in diabetic patients to take action in restoring glucose levels to healthy values in real-time. Diabetic bodies cannot automatically offset the rise and fall of glucose concentrations, correlated to nutrition, insulin production, and fitness. For instance, eating sessions and hormonal oscillations influence glucose mobilization and metabolism. Metabolism capacity of organs, stress events, and the administration of drugs may directly or indirectly affect glucose availability and consumption. Hypoglycemia (low glucose levels) might acutely endanger neuronal cell viability which is a life-threatening condition, while hyperglycemia (high glucose levels) may cause diabetic ketoacidosis and hyperosmolar in the short-term, and in the long-run, permanent vascular and neurotoxic damages. Continuous glucose monitoring, along with the quick normalize for any glucose level deviations might significantly enhances diabetic health through minimizing the hypo- and hyperglycemic episodes that compromise homeostasis.

The literature reviewed in this article was searched on PubMed, Scopus, ScienceDirect, and Google Scholar, for the period between 2003 and 2022. The used keywords were, ‘smart,' ‘contact lenses', and ‘glucose monitoring'. Here, we present an overview on the progress in smart contact lenses developed for continuous glucose monitoring. First, the history, materials, and design of contact lenses are summarized. The composition and function of the tear fluid are further discussed, with particular attention given to tear biomarkers and their correlation with blood counterparts. The different classes of glucose sensors integrated into contact lenses are described in details including the sensor's working principle and properties. The latest advances in smart contact lenses for glucose detection are highlighted.

## Contact Lenses

**Figure 1:**
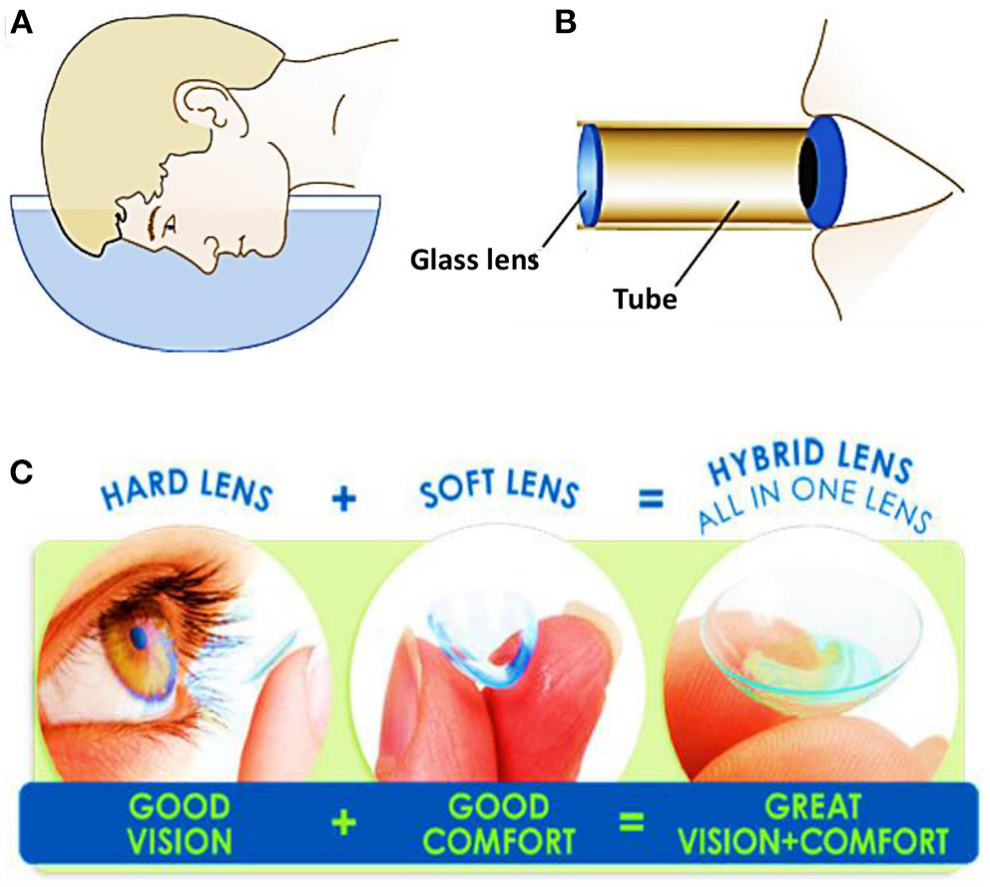
Schematics showing the proposed methods for vision correction: **(A)** Leonardo da Vinci concept: a man looking through a bowel filled of water, and **(B)** René Descartes concept: a man looking through a tube filled with fluid. **(C)** Categories of contact lenses based on the constituents materials.

A contact lens is a thin curved layer of a soft or a rigid transparent material and it is worn in direct contact with the cornea. Contact lenses are obtained from transparent materials, and are sometimes slightly tinted to make them easier to handle. Although contact lenses may seem a modern invention in eye-care, they have a long development history starting from the sixteenth century (6). In 1508, Leonardo da Vinci produced sketches proposing optical solutions for vision correction. He demonstrated that looking through the bottom of a glass bowel filled with water could help rectifying the vision. However, his suggestion was impractical (Figure 1A) (7). Later, in the seventeenth century, René Déscartes introduced the concept of using a test tube filled with water to achieve similar results (Figure 1B). The suggestion of using a tube instead of an entire bowel of water was simpler, but still impractical. The first contact lenses were made of blown glass in 1887 and they were designed such to cover the whole eye surface (7). Although these lenses were beneficial for vision correction, they brought to light several other points with a space for improvement. Firstly, glass lenses were heavy on the eye for long-term wear. In addition, thick glass is not oxygen-permeable, making these lenses harmful for the eye cornea when worn for few hours. In 1940s, hybrid contact lenses made of glass and poly (methyl methacrylate) (PMMA), were introduced. The glass portion covered the cornea, and the sclera was covered by the polymer portion. The lenses were lighter and allowed more oxygen to permeate. The lens design was further brought to the next stage, where the lens covered only the eye cornea and the sclera was left free to breathe naturally (Figure 1C). Non-porous, thin PMMA allowed oxygen to reach the cornea only during blink where the lens moves, and subsequently the lens could be worn for longer periods without causing irritation. A massive breakthrough in contact lenses manufacturing was made by the Czech chemist Otto Wichterle who made the first soft hydrogel contact lens. The lens was made of poly (2-hydroxethyl methacrylate) which is transparent, capable of absorbing water up to 40% of its weight, and could be molded into a more anatomical shape (Figure 1C) (8, 9). In early 1970s, the first commercial soft contact lenses were released after being approved by the Food and Drug Administration (FDA). Since then, soft contact lenses were subjected to developments to increase their oxygen permeability and water absorption properties. In the late of 90s and early 2000s, silicon hydrogel became the material of choice for most contact lens manufacturers (10). Silicon hydrogel lenses allowed up to five times higher oxygen permeability, which helped to keep the eye hydrated, healthy, and comfortable for longer times than before.

Commercially available contact lenses can be classified into three categories according to their constituent materials: (i) soft contact lenses such as poly-HEMA, polyacrylamide (PA), polyethylene terephthalate (PET), poly(vinyl) alcohol (PVA), and polydimethylsiloxane (PDMS), (ii) rigid contact lenses made of hard polymers such as PMMA, and (iii) hybrid contact lenses, which combine soft and rigid materials, i.e., a center rigid gas permeable portion with an outer skirt made of a soft contact lens material. Among these contact lenses, soft lenses are the most commonly used in the integrated diagnostic technologies, because of their high oxygen permeability along with the user comfortability and the usage prevalence (11). Characteristics of the three categories of the contact lenses are summarized in Table 1, along with some selected commercial models. Advantages, disadvantages, polymer materials, equilibrium water content EWC%, supplier, and the commercial name (C.N) of the contact lenses are provided.

**Table 1:** Comparison among the different categories of contact lenses and some selected commercial contact lenses (9).

**CL type**	**Advantages**	**Disadvantages**	**Polymer**	**EWC (%)**	**Supplier**	**C. N**.
Soft hydrogel CLs	i. Increased initial comfort ii. Require short period for adaptation iii. More stable on the eye cornea iv. Less probability that tiny particles to stick in between the lens and cornea	i. Worn for short period compared to hard lenses ii. Have less gas permeability compared to hard lenses iii. provide less sharp vision iv. Induce more allergic problems v. May cause eye dryness vi. Can easily break	HEMA	38	Bausch & Lomb	Polymacon
			PVA	69	Alcon	Dailies AquaComfort plus
			HEMA, DAA, MAA	45	CIBA Vision	Soft Mate II
			HEMA, MAA	58	Johnson & Johnson	1-d Acuvue moist
			HEMA, MAA	55	Coopervision	Frequency 55
Silicon soft CLs	i. High oxygen permeability ii. Reduce hypoxia-related symptoms iii. Allow extended wear	i. Not suitable for people with silicon intolerance ii. Collect more deposits iii. Less comfortable on delicate eyes	Alkyl methacrylates, siloxane, NVP	56	Sauflon	Clarity 1 d
			SIMA, SIA, DMA, pyrolidone derivative	40	Menicon	Premi
			NVP, VMA, IBM, TAIC, FM0411M, HOB	48	Coopervision	Biofinity
			NVP, TPVC, NCVE, PBVC	36	Bausch & Lomb	Pure Vision
Hard CLs	i. Offer sharp vision ii. Deposit resistance iii. Durability iv. Cost-effective v. Better option for sufferers of astigmatism	i. Require a period for adaptation ii. Require regular usage for comfort iii. Easily to move out of the eye center iv. Require daily cleaning	Fluorosilicone acrylate	N/A	InnoVision	Accucon
			Silicone Acrylate	N/A	Bausch & Lomb	Boston II
			Fluoro-Silicate Acrylic	N/A	G.T Laboratories	Fluorex 300
			Fluorosilicate Acryle	N/A	Contamac	Hybrid FS
			Fluoro-Siloxayanyl Styrene	N/A	Menicon	Menicon Z
Hybrid CLs	i. Increased oxygen permeability ii. Easy to handle iii. Quick adaptation iv. Provide a stable vision quality iii. Easy care	i. More careful evaluation of lens-to-anterior segment required ii. Costly iii. Require time to settle iv. Difficult to apply and remove	Silicone hydrogel/RGP	50% for the silicone hydrogel skirt	EyeBrid Silicone	Eyebrid
			Silicon hydrogel Filcon V3/roflufocon D	50% for the silicon hydrogel skirt	SwissLens	AirFlex

### Contact Lens Design

In designing contact lenses, different parameters must be considered. Contact lenses have to be fabricated with base curve radius (BCR) in the range of 8–10 mm to fit comfortably in the eye cornea, and to facilitate tear exchange and oxygen permeability (12, 13). Contact lenses are produced with a diameter range of 14–15 mm to fit different eye sizes easily. The central thickness (CT) of the contact lens is a significant parameter, as it influences the amount of oxygen permeating the lens and further reaching the cornea. Most commercial contact lenses are produced with CT of about 0.1 mm (14). The optical power of the contact lens is the main responsible factor for vision correction, and it measures the degree to which the lens converges the light. The lens optical power is given by the reciprocal of the focal length and its SI unit is inverse meter (m^−1^), which is called diopter (15). Worn contact lenses are subjected to stresses resulting from eye movements and repeated usage, which might cause irreversible deformation or fracture, leading to deterioration of optical performances and comfort. Hence, the mechanical properties of the contact lens, such as Young's modulus and tensile strength, have to be optimized (16). The transparency of the contact lens material that allows the incident light to go through the lens to be focused in the eyes is a significant property, besides the relatively high refractive index. Chemical properties of the contact lens material such as water content, free-to-bound water ratio, and biological inertness, also affect contact lens performance (17, 18).

### Tear Fluid and Biomarkers

Intense investigations have been carried out for decades to find an alternative non-invasive body fluid that could replace blood sampling in clinical settings. Examples include interstitial fluid, tears, salvia, and urine. However, alternative fluids could not provide a reliable alternatives in many cases. However, a successful example of blood replacement was found in the context of bladder cancer screening (19), where urine is the ideal sample for diagnosing due to its direct contact with the tissue (20). Eye basal tear fluid is a multilayered structure containing enzymes, proteins, lipids, and electrolytes (6). Basal tears count three layers which serve to protect, clean, and lubricate the eye. In addition, the eye produces psychic and reflex tears resulting from laughing/crying and irritating conditions, respectively. Tears and blood are separated by a barrier, which makes a compositional difference between blood and tear fluid (21). However, the blood supplies the brain passes through this barrier, causing the leak of metabolites from blood to the tears. Consequently, blood-tear correlations were established as the basis to develop tear proxies that could mirror the blood levels of some metabolites (Table 2) (36–38). It was demonstrated that concentration of glucose, lactate, Na^+^, K^+^, Mg^2+^, Ca^2+^, Cl^−^, and urea are correlated with their counterparts in blood (38–40). Additionally, the number of proteins detected in eye tears was in the range of 54–1,543, but the protein figure was found to be strongly dependent upon the sampling method (41). Researchers are investigating the tear fluid for healthy people and cancer patients to establish a relationship among the biomarkers and the different types of cancers. For instance, high levels of lacryglobin were reported for breast cancer patients. Also, lacryglobin was detected in tears for patients suffer lung, colon, prostate, ovarian, and breast cancer (42–44). Several breast cancer biomarkers were detected in eye tears such as protein S 100A8 and triosephsphate isomerase as the concentration of these biomarkers were different in cancer patients compared to healthy controls. L-lactate concentration in tear fluid was found to be correlated with different types of cancer (23). In fact, many types of cancers were found to share the same biomarkers, pinpointing that diagnosing a specific type of cancer based on tear analysis only is a challenge. However, it is noteworthy to measure the susceptibility of cancer at early stages.

**Table 2:** Comparison between tear and blood biomarkers, and their applications.

**Analyte**	**Tear Con. (mM)**	**Blood Con. (mM)**	**Diagnostic disease**	**References**
Glucose	0.01–0.05	3.3–6.5	Diabetes	(22)
Lactate	2.0–5.0	0.36–0.75	Cancer, sepsis, ischemia, and liver disease.	(23)
Urea	3.0–6.0	3.3–6.5	Renal function	(24)
Dopamine	0.37	475 ×10^−9^	Glaucoma	(25)
Total protein	7 g/L	7 g/L	Dry eye syndrome	(26)
Pyruvate	0.05–0.35	0.1–0.2	Genetic disorders of mitochrondrial energy metabolism	(27)
Ascorbate	0.22–1.31	0.04–0.06	Diabetes	(28)
Na^+^	120–165	130–145	Hypo/hypernatremia	(29)
Cortisol	1–40 ng/ml	100–200 mcg/ml	Stress levels and brain injuries	(30)
Cl^−^	118–135	95–125	Hyper/hypochloremia	(31)
Ca^2+^	0.4–1.1	2.0–2.6	Hyper/hypocalcemia	(32)
HCO${}_{3}^{-}$	20–26	24–30	Respiratory quotient indicator	(33)
Mg^2+^	0.5–0.9	0.7–1.1	Hyper/hypomagnesemia	(34)
K^+^	20–42	3.5–5.0	Hyper/hypokalemia and an indicator for ocular disease	(35)

Contact lenses which are in direct contact with eye tears can be functionalized or integrated with tiny sensors to continuously detect different metabolites for diagnostic applications. For instance, smart contact lenses for continuous glucose detection is under development by Inwith Corporation, and another contact lens for glaucoma monitoring has been recently approved by the FDA.

### Advantages of Using Contact Lenses as Wearable Medical Diagnostic Devices

Conventional diagnostic devices require blood or serum sampling which is considered invasive, painful, inconvenient, and accompanied by the risk of infection. Additionally, most of these devices are complicated to handle, costly, and bench-top, hence they cannot be used at point-of-care settings. Moreover, they are not designed for continuous sensing over 24-h, due to the inaccessibility of the sample. However, multiple diseases would greatly benefit from continuous monitoring technologies, such as diabetes and glaucoma. In contrast, contact lenses combine many features that make them ideal as medical devices for biosensing applications (45–51). In the United States, 45 million people were relying daily on contact lenses in 2016, and this figure has been increasing over time, reflecting the popularity of contact lenses (52). Additionally, contact lenses provide physical contact with eye tears and human tissues for long periods. Contact lenses are small in size, light in weight, cost-effective, portable, and are considered minimally-invasive devices. with the capacity of incorporating an assortment of sensors. The intimate relationship among the eye parts and contact lenses gave possibility to develop lenses to function as continuous monitoring platforms.

## Smart Contact Lenses for Continual Glucose Detection

As mentioned earlier, the tear fluid composition has a close relationship with the one in blood, due to plasma leakage from blood into tears *via* the blood-tear barrier (35, 37, 38). Recent studies show that glucose levels in tears correlated with blood glucose; however, the glucose levels in tears were found to delay for 10–20 min (53). Smart contact lenses developed for glucose detection are classified into three categories based on the type of the sensor integrated with the contact lens. This section discusses each category, including their working principles, advantages, drawbacks, limitations, and recent advances.

### Fluorescence-Based Contact Lenses for Glucose Detection

Fluorescence occurs when an incident light of a wavelength range 200–800 nm is absorbed by specific molecules, which transit certain electrons to higher energy levels (54). The excited electrons return to their ground states emitting the absorbed light in the form of fluorescence. However, some of the absorbed light is lost in form of heat or vibration. Consequently, the emitted light has lower energy than that of the absorbed. The electronic transition is an instantaneous process lasting 10^−15^ s, and the lifetime of the excited state is around 10^−8^ s. Therefore, the whole process of fluorescence emission lasts about 10^−8^ s (55). The fluorescent molecules (fluorophores) can lose the absorbed light not only by re-emission heat or vibration, but also by transferring the energy into another fluorescent molecule or fluorophore nearby. When part of the excitation energy is transferred from the excited/donor fluorophore to a ground state of an acceptor fluorophore, the process called Förster resonance energy transfer (FRET) (56). The amount of the transferred energy depends on the spectral overlapping between the fluorophores (donor and acceptor fluorophores) and the interspace between them (57). Förster resonance energy transfer is considered as a fluorescence suppression process for the energy absorbed by the donor fluorophore.

Fluorescence-based sensors are synthesized from a certain analyte-receptor/molecular recognition agent, a donor fluorophore, and an acceptor fluorophore, which all lie in vicinity of each other. In the FRET-based sensors, when the analyte binds with the receptor, the receptor undergoes a chemical structural change moving the fluorophores farther apart, which decreases number of electrons transferred to the acceptor fluorophore. The FRET decreases, resulting in an increase in the fluorescent emitted light, which can be correlated to the analyte concentration. An additional mechanism can be used for developing fluorescent sensors: a receptor/molecular recognition agent which competitively binds the biomarker/analyte (58). In this mechanism, the fluorophore binds the receptor, leading to an electron transfer from the fluorophore to the receptor, which causes the fluorophore to emit fluorescent light. However, in presence of the biomarker/analyte, the receptor binds the biomarker separating the fluorophore from the receptor, and because the conduction energy levels of the fluorophore are full, no fluorescence occurs. This means that in this mechanism the higher the biomarker/analyte concentration, the lower the fluorescent emission (59).

**Figure 2:**
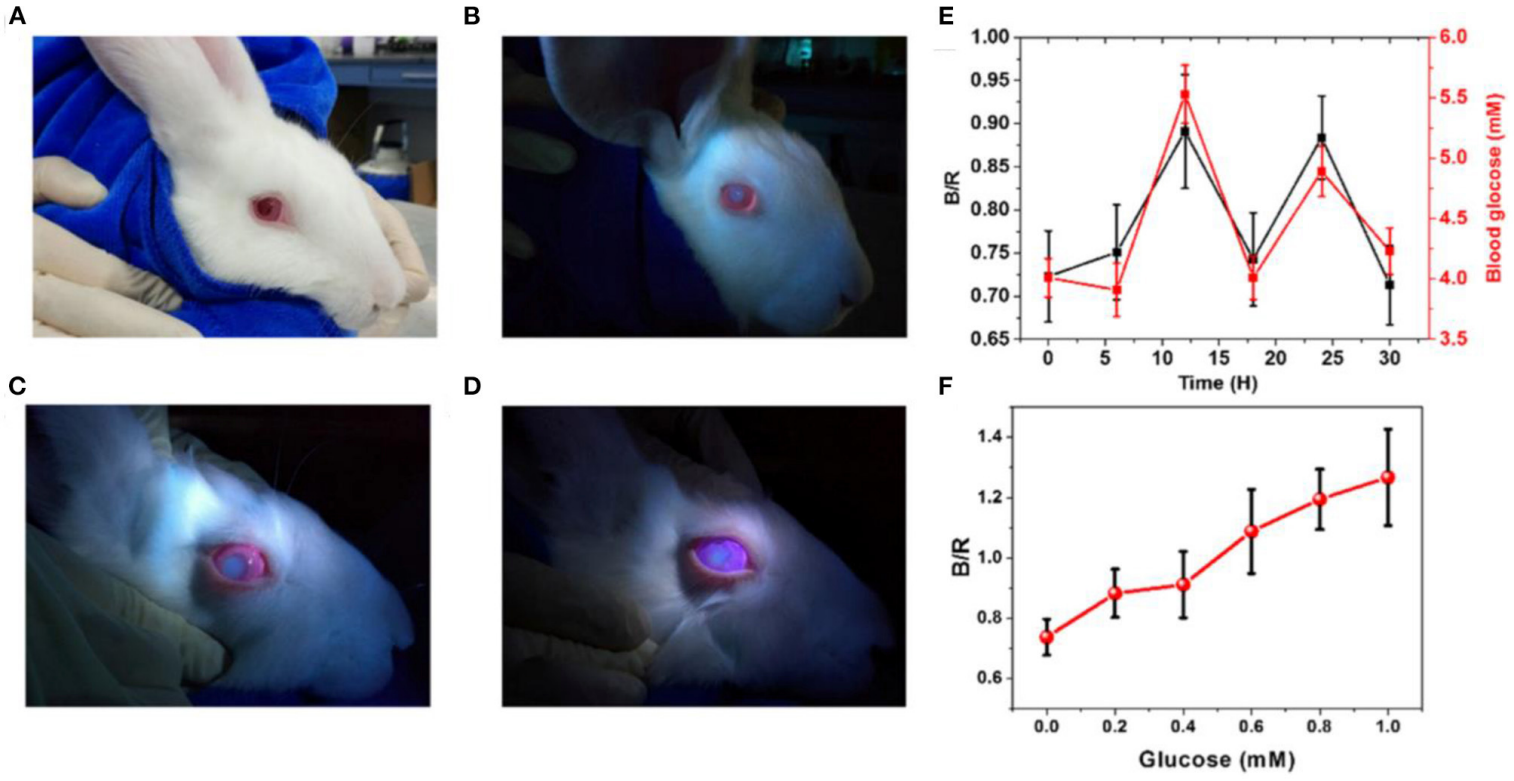
Florescence-based smart contact lens for tear glucose sensing. **(A)** Photographs of the rabbit wearing the smart contact lens under ambient light illumination. **(B)** The rabbit eye wearing contact lens under illumination by UV LED light. **(C)** The worn lens in artificial tears containing glucose of 1 mM concentration. **(D)** The worn lens in artificial tears containing glucose of 5 mM concentration. **(E)** Blood glucose levels measured by a commercial glucose meter over time and their corresponding tear glucose levels measured by analyzing the fluorescent signals emitted from the contact lens worn on the rabbit eye. **(F)** The ratio of the blue and red channel (B/R) for the images taken for the contact lens worn in the rabbit eye and immersed in artificial tears with different glucose concentrations (67).

Fluorescent sensors have been utilized in various applications due to their versatility, sensitivity, and selectivity. Fluorescent glucose sensors were integrated into contact lenses for continuous glucose detection in tear fluid. The first contact lens integrated with fluorescent sensor for glucose tear monitoring was developed by Badugu et al., in 2003 (60). Daily disposal contact lenses were embedded with boronic acid containing fluorophores. The contact lens showed to be suitable for detecting tear glucose levels in the range of 0.05–1.0 mM. The contact lenses showed a reversible response, and functioned in the physiological pH and ionic strength. The same work team developed colorless contact lens for glucose sensor based on a novel boronic acid containing fluorophores, which were compatible with low pH and methanol-like polarity (61). The excitation and emission light for the embedded sensor were in the UV range and the sensor responded to tear glucose concentration in the range of 0.05–0.5 mM. The sensitivity of the sensor was 20% for glucose change from 0.05 to 0.5 mM, and the response time was 10 min. Recently, the same research team reported a methodology for tear glucose monitoring based on silicon hydrogel contact lenses (SiHG) (62). The glucose recognition agent, fluorophore Quin-C18, strongly bounded to the lens without showing any significant leaching after multiple rinses. The contact lens was tested *in-vitro* and showed a robust performance as similar response to glucose after 3 months of storage, was observed. March et al. developed fluorescent glucose sensors based on a fluorophore and a glucose recognition agent trapped in hydrogel spheres that were inserted into a soft contact lens made of poly (vinyl alcohol) (63). When the glucose molecules reacted with the glucose receptor, the fluorophores shifted away from the receptor, decreasing FRET and increasing the fluorescent light upon increasing glucose concentrations. However, a 15 min delay in the glucose concentration readouts was observed. The sensor was compatible with a hand-held fluorometer, used to collect the output signal. Moreover, pHEMA and PDMS contact lenses have been integrated with fluorescent glucose sensors. The glucose-responsive molecules and the organic fluorescent dye were encapsulated in silica nanoparticles, and were embedded in the contact lenses. The silica retained the capsule shell integrity and prohibited the leakage. The contact lenses were able to detect glucose concentration in the range of 0.5–5.0 mM (64). Also, daily disposal commercial contact lenses were integrated with fluorescent glucose sensors based on boronic acid-containing fluorophores. Glucose concentrations in the range 0.05–0.50 mM were detected with an equilibrium time of 10 min. The pH, chloride, and polarity were found to be interfering with the sensor readings (65, 66). Recently, a highly sensitive fluorescent glucose sensor together with a reference probe were implemented into pHEMA contact lens to achieve ratiometric analysis with obvious fluorescence color changes, for the colorimetric detection of glucose concentrations in eye tears (Figures 2A–F) (67). The glucose levels in tears were monitored *via* a smartphone as a reader, which worked by capturing and analyzing the fluorescent images of the contact lenses. An increase in glucose concentrations led to a shift in the fluorescent color of the contact lens from pink to blue. The contact lens detected glucose concentrations in the range of 0.23 μM-1.0 mM, in artificial eye tears. The biocompatibility of the contact lenses were demonstrated in the tests carried out on animals. More recently, Deng et al. reported soft contact lenses which could detect glucose in tears with high sensitivity (67). A glucose fluorescent recognition molecule probe and a reference fluorescent dye formed the glucose sensor that was immobilized into the hydrogel matrix of the contact lens. With increasing glucose concentration, the color of the contact lens changed from pink to blue due to shifting the fluorescent color. The detection system of the sensor's signal was replying on a smartphone camera that capture photographs for the contact lens and a software transformed the images into RGB signals to quantify the glucose levels. The sensor showed a working range of 0.023–1.0 mM, and biosafety when it was tested on animal models (67).

A variety of fluorescent sensors were developed and integrated with contact lenses to monitor many other biomarkers in tears. For example, Lakowicz and his group developed ion sensors (Cl^−^, K^+^, Na^+^, Ca^2+^, and Mg^+2^), and integrated them into silicon commercial lenses for long-term detection of dry eye syndrome (68). These proof-of-concept studies paved the way for more fluorescent probes to be coupled with contact lenses and utilized for monitoring biomarkers, such as lactate, potassium, magnesium, sodium, and urea.

Fluorescent sensors have been utilized in various applications due to their versatility, sensitivity, and selectivity. However, they suffer from high intensity background fluorescence which commonly exists in biological media. The fluorescence-based molecular recognition agents are influenced by the ambient oxygen concentration and temperature. The fluorophores have low chemical stability due to photodegradation of the optical species (69). Furthermore, some fluorescence receptors require the presence of solvents to function (70), which may be challenging to be integrated in a wearable lens.

### Electrochemical Contact Lens Sensors for Glucose Detection

Electrochemical sensors have been developed for medical diagnosis over the last few decades. They can be classified into two categories based on their response to glucose: (i) selective, and (ii) non-selective. Only the glucose selective electrochemical sensors lies in the scope of this review. Semiconductor fabrication techniques were employed for producing the electrochemical sensors (71). Generally, electrochemical sensors are constructed from three electrodes: reference, working, and counter electrodes. Selective electrochemical sensors are manufactured for detecting various biomarkers such as glucose, lactate, uric acid, cholesterol, dopamine, and drugs (72–75). For selective glucose detection, enzymes offer some superior advantages in terms of selectivity, sensitivity, and rapid response. For example, glucose oxidase is used in glucose detection where the enzymatic reaction converts glucose into gluoconolactone and hydrogen peroxide. Hydrogen ions, oxygen, and electrons come out of the dissociation process for the produced hydrogen peroxide. The generated electrons are utilized by the three-electrode system to measure the glucose concentration.

**Figure 3:**
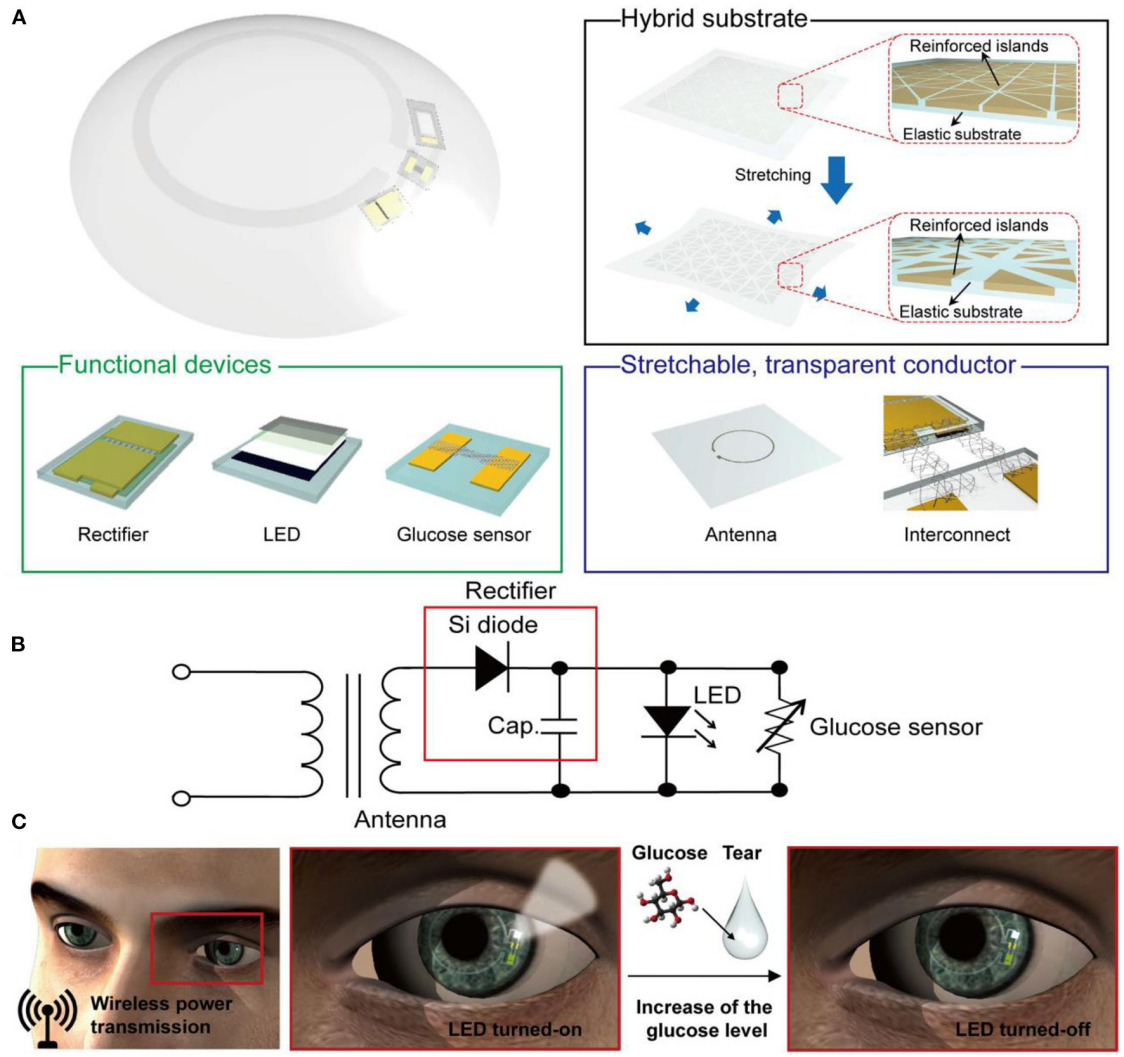
A stretchable transparent smart contact lens system. **(A)** Schematic diagram of the soft contact lens; the lens is composed of a hybrid substrate, functional devise (rectifier, LED, and glucose sensor), and a transparent stretchable conductor (for antenna and interconnects). **(B)** A diagram of the circuit used in the smart contact lens system. **(C)** Operation of the smart contact lens; the electric power is wirelessly transmitted to the lens through the antenna, which in turn activates the LED pixel and the glucose sensor. The pixel turns off once the detected glucose level exceeds the normal limit (80).

Recent advances in transparent electrodes, wireless communication technology, and biosensors have accelerated the progress of smart contact lenses. Extensive research efforts were carried out by Google and Novartis to develop contact lens sensors that can be powered wirelessly and provide glucose concentrations in the tear continuously. Soft contact lenses for glucose measurements in tear fluid have been developed by integrating electrochemical sensors with contact lenses (76). For instance, Yao et al. developed PET contact lenses for continuous glucose detection based on a three-electrode sensor made of titanium (Ti, 10 nm), palladium (Pd, 10 nm), and platinum (Pt, 100 nm). Thin films were sequentially deposited by thermal evaporation onto the wafer, to form the electrodes. Immobilization of glucose oxidase was achieved through a deposited film of titanium oxide which also acted as a passivation layer for the contact lens surface. Potential interferences of urea, ascorbic acid, and lactate were minimized by using Nafion film. In order to test the sensor performance, electrical connections were defined by three large pads of 2 mm^2^ each, and the current flow through the working electrode was recorded. The sensor exhibited a fast response time of 20 s, and a limit of detection lower than 0.01 mM. A sensitivity of 240 μA cm^−2^ mM^−1^ was recorded in the glucose concentration range of 0.1–0.6 mM. The same group reported PDMS contact lenses integrated with another type of electrochemical sensor for glucose detection in tears (77). The sensor combines three electrodes made of platinum, silver, and silver chloride. The fabrication of the sensor was carried on a PDMS layer of thickness 70 nm that later was bended to conform to a contact lens shape. Sputtering techniques were used to deposit the electrodes: 200 nm of Pt layer, and 300 nm of silver layer. GOD was immobilized on the electrodes through trapping it in polypropylene membranes. For *in vitro* glucose testing, the contact lens-integrated sensor measured glucose concentrations in the range from 0.03 to 5.0 mM. The *in situ* testing was carried out by placing the contact lens onto the eyeball of a rabbit, and the results were correlated with the outputs given by conventional blood glucose readers. Glucose concentrations in both blood and tear were peaked following the oral intake of glucose. The tear glucose showed a delay of 15–20 min compared to the glucose levels in blood, and both reached the maximum after 55 min of glucose intake. The contact lens showed a linear relationship between glucose concentrations and the recorded current in the range of 0.025–1.475 mM, with a correlation coefficient of 0.99. The highest sensitivity was achieved at a pH of 7.0, and it was temperature-dependent. The decrease in current density beyond pH 7.0 and 45°C was attributed to the degradation of GOD in the alkaline medium, and to its thermal deactivation, respectively. Also, M. Chu et al. developed PDMS contact lenses for *in situ* glucose monitoring based on GOD enzyme deposited on flexible electrodes (78). The sensor showed a linear relationship with a correlation coefficient 0.99, between glucose levels and the output current in the glucose concentration range of 0.03–5.0 mM The contact lens sensor was tested on a rabbit model and detected its tear glucose levels. Recently, Kim et al. developed a graphene field effect transistor combined with an antenna to function as a glucose sensor. The sensor was integrated into a soft contact lens and operated wirelessly (79). The contact lens was tested for glucose quantification *in vitro* and *in vivo*. Glucose oxidase was chemically attached to the pyrene linker, and was further immobilized on the graphene channel. The signal receiver was placed at 10 mm from the contact lens to collect the antenna signals. More recently, a wireless electrochemical glucose sensor with display pixels was incorporated into a soft contact lens (Elastofilcon A) for monitoring glucose concentration in tears (Figure 3) (80). For the first time, a display was introduced in smart contact lenses to eliminate the necessity of bulky equipment used in signal detection. The performance of the lens was tested on a rabbit eye for glucose concentration in the range of 0.1–0.9 mM. Upon increasing the glucose concentration above the normal levels, the display turned off. Jeon et al. (81) reported polyethylene terephthalate contact lenses-integrated with 3-electrode electrochemical glucose sensor. The electrodes were composed of gold, titanium and silver chloride. The designed IC was implemented in a 0.18 CMOS processor. The RF transmitter and the thin film rechargeable battery were integrated with the contact lens. The system was tested *in vitro* for glucose detection in the glucose range of 3–25 mg/dL, and showed a linear response. K. Hayashi et al. developed the first self-powered contact lenses for continuous glucose monitoring by combining the transmitter with a glucose fuel cell that functioned as both the power source and a sensing transducer (82). The self-power operation of the contact lens was verified in a glucose concentration of 30 mM. Recently, the first solar cell powered contact lenses for glucose monitoring, was reported (83). A LED was implemented in the contact lens to eliminate the necessity for wireless communication and the power supply on-lens solar cell eliminated the necessity for wireless power delivery, enabling a stand-alone operation under room-ambient light. Besides glucose sensing alone, smart contact lenses were also developed as drug delivery systems to allow insulin release (84). For instance, Keum et al. (84) reported soft contact lenses for simultaneous glucose detection and insulin delivery. The contact lens was built on a biocompatible polymer containing ultrathin flexible electrical circuit, microcontroller chip, drug delivery system, wireless power management, and data communication apparatus. The contact lens was tested on rabbit models and was validated by conventional blood glucose tests. The drugs were electronically triggered to be released from the reservoirs for bringing glucose levels back to normal levels.

**Figure 4:**
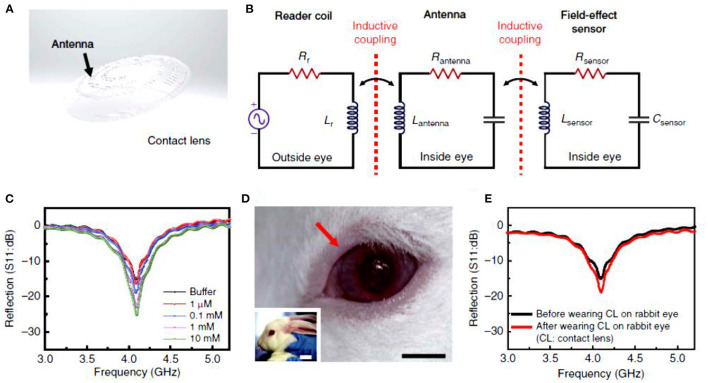
Smart contact lenses for wireless glucose detection. **(A–E)** Electrochemical sensor integrated with a contact lens: **(A)** schematic illustration of the transparent glucose sensor integrated in the contact lens, **(B)** schematic illustration of the reading circuit in the wireless sensing mode, **(C)** wireless glucose measurements in the glucose range of 1–10 mM, **(D)** photographs of the smart contact lens placed on rabbit eyes, and **(E)** wireless glucose detection by the smart contact lens *in vitro* and *in vivo* (79).

Multifunctional contact lens sensors that can monitor glucose along with other physiological parameters were introduced. For instance, smart sensors were integrated with soft contact lenses for wireless detection of glucose and intraocular pressure, simultaneously (Figure 4) (79). Highly transparent and stretchable sensors that can monitor glucose and intraocular pressure were incorporated into soft contact lenses. The breakthrough in this study was the introduction of the hybrid structure of 1D and 2D nanomaterials which added reliability, robustness, flexibility and transparency. Among the incorporated electronic *RLC, R* responded for the glucose concentration whereas *L* and *C* varied with the intraocular pressure. The change in the reflection coefficient due to *R* changes, and the shift in the resonant frequency resulting from *L* and *C* changes, were independent of each other. The contact lenses were tested *in vitro* and *in vivo* for detecting glucose in the range 0–10 mM. Another multiplexed contact lens sensor was developed for simultaneously monitoring glucose and corneal temperature (85). The smart contact lenses relied on ultrathin MoS_2_ transistors and gold nanowires. The sensors were incorporated directly on the surface of PDMS contact lenses to leave the sensors in direct contact with the tear fluid and the ocular surface. *In vitro* studies showed that the sensor system was biocompatible. Continuous glucose monitoring investigations were carried out *in vitro*, in the glucose concentration range of 0.0–0.6 mM, and the measured electric current vs. glucose concentrations showed a linear relationship. The recoded sensitivity in the whole tested glucose range was 45%. The potential interference from the tear ingredients were negligible as the sensor showed the same response in the buffer and artificial tears. The sensitivity for temperature was recorded in the temperature range of 30–50°C and the resistance of the gold nanowires showed a linear trend with the temperature in the tested range. This system provided a novel insight into designing multifunctional bioelectronics sensors. Further, the fabrication strategy allows for embedding other functioning components, such as electrode arrays for electro-retinograms, antennas for wireless communications, thin film batteries/super capacitors, and wireless radiofrequency transmitters. Such multiplexed contact lenses holds great promise in next-generation ocular diagnostics.

However, many challenges still have to be overcome to achieve usability in clinical settings (86). Electrochemical sensors are based on enzymes, which are unstable by nature and suffer degradation in a short lifespan, which limit the employability of the sensor for long periods. Furthermore, the operation conditions such as ambient oxygen, pH, temperature, and humidity influence the sensor performance. Additionally, sterilizing the smart contact lens according to health regulations may lead to enzymes denaturation. The interaction of the hydrogen peroxide with tear ingredients like ascorbic acid interferes with the sensor response (87). The microfabrication of the electrochemical sensor on polymer substrates is also a challenge due to the limitations of the thermal and mechanical properties of the polymer material (88). Electrochemical sensors require an applied power to drive the chemical reaction, which makes the fabrication more challenging. Mostly, electrochemical sensors are made of opaque electronic materials, metal antennas, interconnects, and integrated circuit chips which may block the user vision, and decrease the oxygen permeability (77, 89).

### Light Diffractive Contact Lenses for Glucose Detection

Photonic crystals (PCs) are made of periodically ordered materials of different refractive indexes, and they are classified according to number of periodicity directions into 1D, 2D, and 3D (86). When a photonic crystal is illuminated by a polychromatic light beam, the diffracted light follows Bragg's law:


(1) $$m\lambda_{d}=2n_{eff}\Lambda \;sin(\theta_{d})$$

where *m* is the diffraction order, λ_*d*_ represents the diffracted wavelength, *n*_*eff*_ is the effective refractive index of the PC, θ_*d*_ is the diffraction angle, and Λ is the periodic constant of the PC. Accordingly, the diffracted wavelength changes due to any modification in the periodic constant or in the effective refractive index. Based on this principle, 1D, 2D, and 3D PCs have been developed for sensing applications. When the periodicity Λ of the PC is comparable to the wavelengths of the incident visible light, the PC diffracts a visible color that can be seen by naked eyes. Hence, PC-based sensors exhibit a visible color change. Therefore, PC sensors are strong candidates to be employed in point-of-care settings.

Unlike the selective electrochemical glucose sensors, most of the photonic glucoses sensors are based on phenylboronic acid (PBA) derivatives which are used as glucose recognition agents instead of enzymes used in electrochemical sensors. PBA derivatives form reversible covalent bonds with *cis*-diol molecules and α-hydroxyl acids, such as glucose and lactate, respectively (90). Studies showed that 1D PC (1D-hologram gratings) can be fabricated in many natural and synthetic hydrogel matrices, and can be functionalized with the appropriate receptors to detect a variety of analytes (91). For instance, 1D-PC glucose sensors were developed with polyacrylamide functionalized with 4-vinylphenylboronic acid (4-VBPA). The sensors were prepared by the diffusion method along with the laser interference technique. Complexation of glucose and boronic acid moieties immobilized in the hydrogel network caused volumetric shifts of the hydrogel matrix, inducing a change in the periodic constant Λ, and subsequently the diffracted wavelength/color (91). The main concern was that the sensor could function at a high pH only, above physiological values (92). To develop 1D-PC glucose sensor operates in the physiological pH range, Kabilan et al. used an alternative phenylboronic acid derivative called 3-(acrylamido)phenylboronic acid (3-PBA) (91). The ability of the sensor to bind with glucose under physiological conditions was attributed to the lower p*K*_a_ (dissociation constant) of the 3-PBA, which resulted from the *meta*-position of the acrylamide group on the phenyl ring. The sensor exhibited a visible and reversible response to glucose concentrations under physiological conditions. The sensor appeared green in the glucose-free buffer (PBS) and when exposed to the glucose solution, the diffracted light shifted toward longer wavelengths. The glucose molecules diffused into the hydrogel sensor to bind with the pendant boronic acid groups, resulting in a decrease in the p*K*_a_, and allowed to reconfigure the boronic acid groups to form the charged tetrahedral phenyl boronate anion (93). These charged anions generated Donnan's potential that induced an osmotic pressure, resulting in swelling the hydrogel matrix (94). The swelling was reflected on the diffracted wavelength which red-shifted due to increasing the periodic constant of the sensor. Upon removing the glucose solution and washing the sensor in PBS buffer, the sensor returned to its original volume, hence the diffracted spectrum returned to its original basal diffracted wavelength. Since *cis*-diols binding with boronic acid is reversible, washing out the glucose decreased the concentration of the boronate anions, leading to shrinkage of the sensor due to the elastic restoring force of the hydrogel network that expelled the absorbed water and counter-ions (95). The 1D-PC glucose sensor showed a sensitivity of 18 nm mM^−1^ in the glucose range of 0–11 mM, and it was more selective for glucose over lactate. Later, this sensor was attached to a PVA contact lens for clinical testing (96). Toxicity and performance of the contact lens as a non-invasive glucose sensor were examined after sterilization. The sensitivity of the sensor showed a decline, which means that the sterilization procedures negatively impacted on the sensor performance. However, the sensor responded to glucose in a reversible manner, the sensitivity dropped to 4.5 nm mM^−1^, compared to its value of 18 nm mM^−1^ prior to sterilization. The readout was performed using a white light source connected to an optical fiber, and a spectrometer connected to a computer to record the diffracted wavelengths. An alternative practical detection system would be needed, and the sensitivity of the contact lens sensor should be improved for applicability. Another concern about the described 1D-PC sensor is the usage of phenylboronic acid derivatives that binds with other biomarkers such as lactate, galactose, fructose, and mannose. In this regards, another study on the same sensor considered the response and the equilibrium times, showing that the response time for glucose concentrations below 1 mM was around 7 min. However, faster responses were received for higher glucose concentrations. The equilibrium time for the sensor was about 50 and 70 min for high and low glucose concentrations, respectively. The 1D-PC sensors were fabricated by the diffusion method and the laser interference technique, requiring a multistage process of 10 stages (64). Additionally, there may be concerns about the potential interference of the tears ingredients and the long response time.

On the other hand, 3D-PC glucose sensors consist of highly charged nanoparticles (polystyrene or silica) periodically arranged in three dimensions, in a polymer matrix. Asher's group developed 3D-PC glucose sensors based on polystyrene nanoparticles embedded in co-polymerized hydrogel (polyacrylamide-polyethylene glycol) (97). Two phenylboronic acid derivatives were attached separately to the hydrogel post-photopolymerization. The sensors were tested in synthetic tear fluids at physiological pH and ionic strength. Binding the glucose with the boronate anions occurred through the formation of a bis-bidentate cross-link, a 2:1 PBA-glucose complex, which led to a shrink in the hydrogel, resulting in a blue shift in the diffracted wavelengths due to shortening the periodicity constant. The sensor hue changed and was observable by the naked eye, and shifted from red to blue over the physiological glucose concentration range. The selectivity of the sensor for glucose compared to galactose, mannose, and fructose was demonstrated. The sensitivity was approximately 14 nm mM^−1^ and the limit of detection was as low as 1 μM in synthetic tear fluid. The same group promoted the response kinetics of the 3D-PC glucose sensor by copolymerizing *n*-hexylacrylate with a acrylamide, and functionalized the sensor with 5-amino-2-fluorophenylboronic acid (5A-2F-PBA) (98). The sensor showed a response time of 300 s in glucose concentration of 1 mM at pH 7.4 and a temperature of 22°C. However, the response time was shorter at higher temperatures and at higher glucose concentrations. In the physiological temperature (37°C), the response time went down to 90 s for a glucose concentration of 5 mM. The authors recommended attaching the sensor to a contact lens or inserting the sensor under the lower eyelid for achieving the continuous glucose detection. Diabetic patients could determine their glucose concentrations by observing the color of the sensor using a compact device combining a white light source, a mirror, and a color chart. Hu et al. developed near-infrared 3D-photonic crystal sensor for ratiometric sensing of glucose in tears (99). The novel design of the reported sensor was to overcome the slow response and low sensitivity of the 3D-photonic crystal sensors. In the sensor, the crystalline colloidal array (CCA) itself was glucose-responsive and made of poly (strene-co-acylamide-3-acrylamidophenylboronic acid). The CCA was arranged in a positively hydrogel matrix of poly (acrylamide-co-2-(dimethylamino)ethyl acrylate). Unlike the previous sensors 'designs, the arranged particles in the hydrogel matrix were responding to glucose. The glucose sensor diffracted the wavelength of 1,722 nm, whose intensity decreased with increasing glucose concentration over the physiological relevant range in tears. The sensor showed to be able to detect glucose concentration as low as 6.1 μg/dL. The response time was 22 min for glucose concentration 7.5 mg/dL and decreased with increasing glucose concentration. Braun's group introduced a 3D-PC glucose sensor that linearly and rapidly responded to glucose by introducing a volume resetting agent, polyvinyl alcohol (PVA) (100). The acrylamide pre-gel was polymerized with highly charged polystyrene nanoparticles, followed by attaching the PBA derivative to the hydrogel –and the PVA was diffused into the hydrogel to bind with the boronate anions forming crosslinks, which led to shrinking the hydrogel matrix. When the sensor was exposed to glucose, the PBA-PVA crosslinks were superseded with 1:1 PBA-glucose complexes, resulting in swelling of the hydrogel, and subsequently a red-shift of the diffracted light. The sensor covered the clinical relevant glucose range of 2.2–38.9 mM, presenting a maximum sensitivity of 12 nm mM^−1^ in glucose concentration range of 0–10 mM when 5-amino-2-fluorophenylboronic acid (5A2FPBA) was used as the glucose recognition agent (101, 102). However, the best kinetic response was recorded for the sensor which was functionalized by the derivative 3-PBA, as the time required for the sensor to reach 90% of equilibrium binding was 7 and 12 min for glucose concentration ranges of 0–10 mM and 30–40 mM, respectively. Leakage of PVA from the sensor is expected, which significantly would affect the sensor response/sensitivity and reusability. The highly charged monodispersed polystyrene spheres used in the sensor were synthesized by the emulsion polymerization process, which takes 3 weeks (55, 60). In order to fulfill a rapid fabrication process for the photonic-based glucose sensors, 2D-PC glucose sensor was introduced where a fast fabrication process of 2D-PC at the air/water interface has been reported (103). At the air/water interface, it was possible to produce a hexagonal arrangement of nanoparticles of area 280 cm^2^ in 2 min. The studies showed that these arranged nanoparticles in a monolayer form can be incorporated into stimuli-responsive hydrogels. The forward diffracted monochromatic light passing through the 2D-PC hydrogel showed a Debye diffraction ring of a diameter depends upon the particles interspace, and the Debye ring was exploited to monitor the hydrogel volumetric change. The Debye diffraction follows the formula:


(2) $$\begin{array}{r} \sin\alpha =2\lambda /\sqrt{3}\;d \end{array}$$

where α is the forward diffraction angle of the Debye diffraction, *d* is the interspace among the particles, and λ is the incident wavelength. The forward diffraction angle is given by the relationship;


(3) $$\begin{array}{r} \alpha ={\text{tan}}^{-1}\frac{D}{2h} \end{array}$$

where *D* denotes to the diameter of Debye ring and *h* represents the distance between the sensor and the screen. For instance, Xue et al. developed a 2D-PC glucose sensor based on the air/water interface approach and the sensor was investigated in artificial tears (103). The sensor was selective for glucose over galactose and fructose at the physiological ionic strength (150 mM), and presented short response time (50). However, the sensor was functioning only at high pH values above the physiological pH. Another study reported a 2D-PC glucose sensor which can operate in the physiological pH (104). The sensor showed high sensitivity and a short response time of 180 s. Although detecting the sensor response was simple, the readout methodology is not practical, especially when the sensor is attached to contact lenses. Because the contact lens constrains the sensor expansion/extraction in both x and y directions, leaving the sensor to be free in z-direction only. However, the expansion/extraction in z-direction has no effect on the particles interspace which induces the shifts in the Debye ring's diameter. In addition, measuring the Debye ring diameter in the transmission configuration is not practical in wearable devices. The response of the 2D-PC could not be distinguished by the naked eye, which may limit its applications.

**Figure 5:**
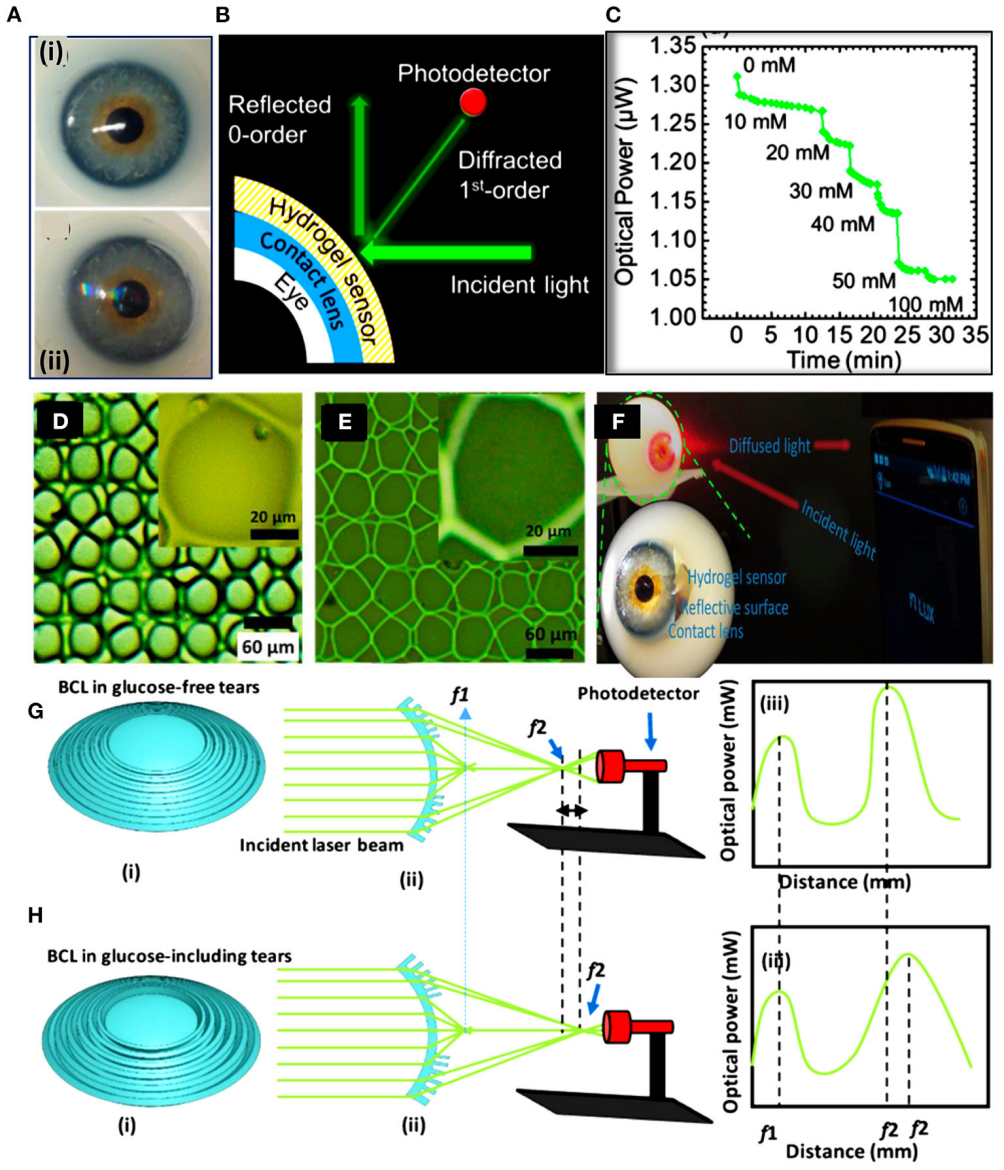
Smart contact lenses for glucose monitoring developed based on light diffractive sensors. **(A–C)** A light diffraction-based glucose sensor integrated with a soft contact lens: **(A)** a soft commercial contact lens placed on an eye ball (i), and the commercial contact lens integrated with a 1D grating glucose sensor (ii), **(B)** schematic illustration of the readout methodology for the contact lens integrated with the 1D grating glucose sensor, and **(C)** continuous glucose detection measurements shows the reflected optical power from the contact lens vs. the glucose concentration (105). **(D–F)** Light diffuser-based glucose sensor: **(D)** optical microscope image for the master holographic light diffuser, **(E)** the surface of the glucose sensor imprinted with the light diffuser microstructures, and **(F)** photographs of the smart contact lens integrated with the light diffuser-glucose sensor and placed on an eye ball to be investigated by a smartphone as a reader (106). **(G,H)** Bifocal contact lenses for glucose detection: **(G)** schematic illustration of the bifocal contact lens in glucose-free buffer, and **(H)** schematic of the bifocal contact lens in glucose-complexation conditions (107).

Recently, Elsherif et al. introduced a smart contact lens for continuous glucose detection under physiological conditions (Figures 5A–C). The hydrogel glucose sensor was made of 3-(acrylamido)phenyl boronic acid immobilized in polyacrylamide hydrogel matrix. A light diffraction element (1D grating) was replicated on the glucose-responsive hydrogel by replica molding. The replicated 1D grating functioned as a transducer to convert the volumetric shift of the hydrogel into an optical signal. The 1D-grating enhanced the surface-to-volume ratio of the sensor and the hydrophobicity, which aided in shortening the sensor response time and saturation time to 3 s and 4 min, respectively, in continuous sensing mode. The glucose sensor was attached to a commercial contact lens for glucose detection in eye tears and a smartphone was used as a reader by exploiting its integrated ‘ambient light sensor'. However, the limit of detection of the smart contact lens was not low enough to work in glucose concentration range of eye tears. Another study by the same group reported smart contact lenses for glucose detection based on light diffusing microstructures (Figures 5D–F). In this study, 3-PBA was used as the glucose recognition agent and the polyacrylamide was the hosting matrix. The light diffusing microstructures were replicated on the glucose-responsive hydrogel to convert the volumetric change of the sensor into a change in the light intensity and the spatial profile of the light beam. A smartphone captured the reflected light signals from the lens and correlated them with glucose concentrations. This study showed that the contact lens sensor can be readout by a white light beam which is more safe than the laser which was used in their previous developed contact lens. At the same time, the light diffusing microstructures imprinted on the sensor kept the advantages of high surface-to-volume ratio and the short response time of the glucose sensor. Recently, bifocal contact lenses were developed for continuous glucose detection and presbyopia (107). The commercial contact lens used for myopia treatment was integrated with a hydrogel glucose sensor. The hydrogel glucose sensor offered a light conversion properties as its surface was imprinted with Fresnel structure. Upon integrating the sensor with the contact lenses, the contact lens offered two focal lengths; one came from the lens curvature which aid in far seeing, and another focal length resulted from the Fresnel structure which assist for near vision and glucose detection (Figures 5G,H). A smartphone was used as the contact lens sensor reader and it was capable of detecting the optical signals of the contact lens sensor, and correlate them with the glucose concentrations. The smart contact lenses operated in the artificial eye tears and showed a linear response trend in the glucose concentration range of 0–10 mM with a sensitivity of 1.5% mM^−1^.

Generally, photonic band gap-based sensors offer inherent merits over their electrochemical counterparts, such as the lack of electrical connections, immunity to electromagnetic interference, relatively easier fabrication process, possibility of remote sensing, and cost-effectiveness (91). Compared to fluorescent glucose sensors, the photonic sensors do not require dyes or fluorophores to operate, eliminating the problem of photobleaching (86).

## Conclusion and Future Prospects

Continuous and real-time monitoring of glucose levels allows for tight control of diabetes early stages and progression. Non-invasive glucose detection is a promising method for improving the life quality and expectancy for diabetics. Optical glucose sensors are under progress, including light diffractive and fluorescent sensors. On the other hand, electrochemical sensors are few steps ahead. Minimizing the electronics and employing transparent conductive polymers in addition to the 3D printing technology may revolutionize electrochemical glucose sensors. As the contact lenses come in direct contact with the eye cornea, significant challenged need to be faced. For example, electric current flow through the electrochemical sensors results in Joule heating, which may affect the temperature of the eye. Hydrogen peroxide is a byproduct of the chemical reaction taking place in the enzymatic glucose sensors, and may cause eye irritation.

Contact lenses initially emerged for vision correction but, recently, novel applications have been unlocked. It might become feasible in the near future to use contact lens sensors to monitor a range of ocular and systemic conditions.

## Author Contributions

ME wrote the original manuscript. RM edited the manuscript. HB led the project and acquired the financial support. FA, AS, and IA assisted in preparing the manuscript and proofread the manuscript. All authors contributed to the article and approved the submitted version.

## Funding

The authors acknowledge Khalifa University of Science and Technology (KUST) and KU-KAIST Joint Research Center for research funding in support on this research (Project Code: 8474000220-KKJRC-2019-Health1). HB acknowledges Sandooq Al Watan LLC and Aldar Properties for the research funding (SWARD Program—AWARD Ref. SWARD-F19-008).

## Conflict of Interest

This study received funding from Sandooq Al Watan LLC jointly with Aldar Properties (SWARD Program—AWARD Ref. SWARD-F19-008. The funder was not involved in the study design, collection, analysis, interpretation of data, the writing of this article or the decision to submit it for publication.

## Publisher's Note

All claims expressed in this article are solely those of the authors and do not necessarily represent those of their affiliated organizations, or those of the publisher, the editors and the reviewers. Any product that may be evaluated in this article, or claim that may be made by its manufacturer, is not guaranteed or endorsed by the publisher.
